# Correction: Genetically engineered gas vesicle proteins with proliferative potential for synergistic targeted tumor therapy

**DOI:** 10.1039/d5ra90006a

**Published:** 2025-01-17

**Authors:** Li Lin, Yan Du, Yaotai Wang, Yong Luo, Fujie Jiang, Haiyan Yang, Li Ren, Jianzhong Zou

**Affiliations:** a State Key Laboratory of Ultrasound in Medicine and Engineering, College of Biomedical Engineering, Chongqing Medical University Chongqing 400016 China zoujzh@cqmu.edu.cn +86-13708302390; b Department of Ultrasound Medicine, Chongqing Shapingba Hospital, School of Medicine, Chongqing University Chongqing 400033 China; c Department of Ultrasound, The People's Hospital of Chongqing Liang Jiang New Area Chongqing 400010 China; d Department of Ultrasound, Chongqing General Hospital, Chongqing University 401147 China

## Abstract

Correction for ‘Genetically engineered gas vesicle proteins with proliferative potential for synergistic targeted tumor therapy’ by Li Lin *et al.*, *RSC Adv.*, 2025, **15**, 157–166, https://doi.org/10.1039/D4RA07532C.

The authors regret the incorrect placement of the following two paragraphs in Section 3.3.

“After single exposure (Fig. S1†), some GVS-E remained viable in the tumor tissue. The percentage of viable GVS-E was between 1 × 10^6^ and 1 × 10^7^ CFU mL^−1^. After double exposure (Fig. S2†), the ablation volume in the PBS group was smaller than in the GVS-E group under the same duty cycle (Fig. S2†). This observation preliminarily assesses the potential for synergistic ablation with multiple exposures of GVS-E, confirming its significant role as a synergistic agent in conjunction with FUAS.

However, factors such as the limited ablation space within animal models, the original parameters' unsuitability for multiple exposures, and overlapping ablation volumes in double exposure, hinder the accurate assessment of ablation efficiency and synergistic effects.”

The above paragraphs should be inserted on page 162 at the end of Section 3.4, after the paragraph ending “… participating in the ablation process significantly optimizes the ablation effect.”

The Royal Society of Chemistry apologises for these errors and any consequent inconvenience to authors and readers.

